# Can subtle changes in gene expression be consistently detected with different microarray platforms?

**DOI:** 10.1186/1471-2164-9-124

**Published:** 2008-03-10

**Authors:** Paola Pedotti, Peter AC 't Hoen, Erno Vreugdenhil, Geert J Schenk, Rolf HAM Vossen, Yavuz Ariyurek, Mattias de Hollander, Rowan Kuiper, Gertjan JB van Ommen, Johan T den Dunnen, Judith M Boer, Renée X de Menezes

**Affiliations:** 1 Center for Human and Clinical Genetics, Leiden University Medical Center, Leiden, The Netherlands; 2 Leiden Genome Technology Center, Leiden University Medical Center, Leiden, The Netherlands; 3 Division of Medical Pharmacology, Leiden/Amsterdam Center for Drug Research, Leiden, The Netherlands; 4 Department of Technology, Study Programme Bioinformatics, Hogeschool Leiden, Leiden, The Netherlands; 5 Pediatric Oncology, Erasmus Medical Center, Rotterdam, The Netherlands

## Abstract

**Background:**

The comparability of gene expression data generated with different microarray platforms is still a matter of concern. Here we address the performance and the overlap in the detection of differentially expressed genes for five different microarray platforms in a challenging biological context where differences in gene expression are few and subtle.

**Results:**

Gene expression profiles in the hippocampus of five wild-type and five transgenic δC-doublecortin-like kinase mice were evaluated with five microarray platforms: Applied Biosystems, Affymetrix, Agilent, Illumina, LGTC home-spotted arrays. Using a fixed false discovery rate of 10% we detected surprising differences between the number of differentially expressed genes per platform. Four genes were selected by ABI, 130 by Affymetrix, 3,051 by Agilent, 54 by Illumina, and 13 by LGTC. Two genes were found significantly differentially expressed by all platforms and the four genes identified by the ABI platform were found by at least three other platforms. Quantitative RT-PCR analysis confirmed 20 out of 28 of the genes detected by two or more platforms and 8 out of 15 of the genes detected by Agilent only. We observed improved correlations between platforms when ranking the genes based on the significance level than with a fixed statistical cut-off. We demonstrate significant overlap in the affected gene sets identified by the different platforms, although biological processes were represented by only partially overlapping sets of genes. Aberrances in GABA-ergic signalling in the transgenic mice were consistently found by all platforms.

**Conclusion:**

The different microarray platforms give partially complementary views on biological processes affected. Our data indicate that when analyzing samples with only subtle differences in gene expression the use of two different platforms might be more attractive than increasing the number of replicates. Commercial two-color platforms seem to have higher power for finding differentially expressed genes between groups with small differences in expression.

## Background

Microarray technologies are now commonly used for genome-wide surveying of gene expression. With the availability of an increasing amount of data from different studies, there is a growing need for comparison and combination of datasets. This would be helpful to increase statistical power and to compare biological processes. Comparisons across different studies are, however, complicated by the use of different platforms. Over the past years, many microarray platforms, based on different technologies, have been developed by commercial and academic institutions. How reliable and consistent the results from different platforms are is still a matter of debate [1-3]. Initially, platforms comparison studies were mainly focused on comparison between commercial chips (mainly Affymetrix) and in-house spotted microarrays [4-7]. In recent years, more comprehensive studies were done, some of them reporting agreement between platforms [8-13] and some of them not [14-20]. The largest comparison was performed within an FDA-initiated program for evaluation of the reproducibility, quality and consistency of microarray platforms (MicroArray Quality Control, MAQC). In general, a high agreement between platforms was reported [21-25]. Our study is an extension to previously published studies in several aspects: we investigated the capabilities of five microarray platforms with high technological diversity to identify differences in gene expression in a challenging and highly controlled biological condition, where the expected level of transcriptional regulation was low, the number of differentially expressed genes small, and the number of biological replicates small, but realistic.

The biological question addressed was the finding of differential gene expression in the hippocampus between transgenic mice overexpressing a splice-variant of the doublecortin-like kinase-1 gene, δC-doublecortin-like kinase (DCLK)-short, which makes the kinase constitutively active [26]. The DCLK gene has recently been implicated in crucial aspects of embryonic cortical development by controlling neurogenesis, neuronal migration and neuronal vesicle transport [27-30]. DCLK-short is not expressed during embryogenesis, is abundantly expressed in adult limbic brain structures, particularly in the hippocampus [26], and has mild kinase activity *in vitro *[26,31]. The biological function of DCLK-short expression in the adult hippocampus is largely unknown and the transgenic mice have subtle phenotypes with no obvious differences in basal outcomes (Schenk et al, in preparation). Microarray-based expression profiling of the hippocampus tissues from δC-DCLK-short and controls should reveal the biological processes in which the gene is involved.

The main aim of this paper is to compare the performance of different microarray platforms to detect differences in gene expression in biologically related samples. The performance of and the consistency between the microarray platforms on the level of affected genes and gene sets are reported here. The biological findings will be discussed in more detail elsewhere (Schenk, in preparation).

## Results

### Experimental set-up

Gene expression in the hippocampus of five wild-type mice and five transgenic mice was evaluated with five microarray platforms (Table 1): Applied Biosystems (ABI), Affymetrix (AFF), Agilent (AGL), Illumina (ILL), and home-spotted oligonucleotide arrays (LGTC). Ten chips were used for each platform. For the two-color arrays, a wild-type sample was always co-hybridized with a transgenic sample and the design was balanced with respect to dye. Platform-specific processing of the signal was kept to a minimum as to not introduce processing artefacts. After careful performance evaluation, different normalization methods were chosen for one and two-color, but within the groups of one- and two-color platforms the method was kept constant as not to introduce differences due to the normalizaton algorithm. Differential gene expression was evaluated with an empirical Bayes linear regression model (EBLRM) from the R package limma [32]. Raw and normalized data are available from Gene Expression Omnibus (GEO) under series GSE8349.

**Table 1:** Description of the platforms under study.

Platform	Name	#Probes	#Unique UniGene IDs	#Ensembl transcripts *	one/two color	# DEGs (10% FDR)	# DEGs UniGene dataset (N = 10,876)	# DEGs Ensembl dataset (N = 12,744)
ABI	Applied Biosystem- AB1700	35,948	19,013	25,858	one	4	4	5
AFF	Affymetrix – Mouse Genome 430 v2.0 Array	45,101	20,320	23,553	one	130	72	112
AGL	Agilent- WMG G4122A	41,232	20,612	25,845	two	3,051	1,594	2,003
ILL	Illumina- Sentrix Mouse- 6 Expression BeadChip	46,133	19,292	25,629	one	54	19	24
LGTC	Home-spotted 65-mer oligonucleotide arrays (Sigma- Compugen collection)	21,997	15,261	18,104	two	13	22	35
All Platforms			10,876	12,744		-	285**	693**

* More probes could correspond to the Ensembl transcript.

** The number of differential genes obtained comes from the integrated analysis of data from all platforms within one statistical model which takes the platform into account (cf. Discussion section)

### Detected transcripts

There was a large difference between the platforms in the number of probes which generated a signal above background. AGL had the highest number of present calls, LGTC the lowest. To make a fair comparison across platforms, we re-annotated all probe sequences and mapped them to the Ensembl transcript database. In addition to providing the most up-to-date annotation, alternatively spliced transcripts are considered separately so that possible inconsistencies between platforms due to measuring different splice variants would be excluded. The number of detectable Ensembl transcripts was high on AGL (22,510), intermediate on AFF, ILL, and ABI (around 13,000) and low on LGTC (2,017) (Table 2). The low number of detectable transcripts on the LGTC platform is mainly due to background problems, causing negative control spots to occasionally give high signals. The overlap between detectable transcripts is highest between AFF and AGL (62%) and lowest for all LGTC combinations.

**Table 2:** Overlap in detectable Ensembl transcripts across platforms. The number of detectable transcripts is presented on the diagonal (bold), with the total number of interrogated transcripts for each platform between parentheses. The overlap in the number of detectable transcripts for each pair of platforms is presented in the right side of the table, with the total number of interrogated transcripts shared between each pair of platforms between parentheses. The pair-wise overlap in detectable transcripts as a percentage of the overlapping set of interrogated Ensembl transcripts is presented in the left side of the table.

Detectable transcripts	ABI	AFF	AGL	ILL	LGTC
ABI	**13331 (22963)**	8897 (15950)	11863 (20858)	9449 (21055)	1557 (15221)
AFF	55.8%	**11683 (18572)**	10986 (17698)	9226 (17645)	1487(13987)
AGL	56.9%	62.1%	**22510 (26233)**	12120 (23933)	1800 (17329)
ILL	44.9%	52.3%	50.6%	**13376 (26550)**	1617 (17225)
LGTC	10.2%	10.6%	10.4%	9.4%	**2017 (18591)**

### Differentially expressed genes identified on each platform

The number of significantly differentially expressed genes (DEGs) detected with a fixed False Discovery Rate (FDR) of 10% greatly varied across platforms (Table 1): 4 probes were selected by ABI, 130 by AFF, 3,051 by AGL, 54 by ILL, and 13 by LGTC. As expected, the observed degree of differential gene expression was small. The absolute expression differences for the DEGs were in the following range: 1.45 – 2.23-fold (ABI), 1.10 – 2.58-fold (AFF), 1.05 – 2.40-fold (AGL), 1.15 – 1.92-fold (ILL), and 1.04 – 1.47-fold (LGTC). The only two DEGs with a more than two-fold change in expression (as found with multiple microarray platforms and confirmed by qPCR) were: *Plac9 *(up) and *Gabra2 *(down).

We further investigated the surprisingly high number of DEGs detected by AGL. When intensities instead of ratios were taken into the statistical analysis, no differential genes were detected at a FDR of 10% unless dye and array effect were included in the model. With the latter model (model 3 in the Methods section), 3,570 genes were selected, among which all the 3,051 genes selected by the log ratios-based analysis. This and the more elaborate evaluation presented in Additional file 1 suggest three major explanations for the good performance of the AGL platform: co-hybridization of samples from the two different biological groups to the same array, doubling of the number of observations with the same number of arrays used for the one-color systems, and low noise levels. These conclusions are in accordance with observations from earlier studies [13,33].

The low number of DEGs on the ABI platform may be partly attributable to the use of different batches of arrays, but including the batch effect in the statistical model did not result in more DEGs.

### Analysis of overlapping DEGs across platforms

To be able to compare results across platforms, we created two data subsets with genes or transcripts interrogated by all platforms. For the first subset all GenBank accessions that were used by the array suppliers for their probe design were mapped to Unigene (UG) database, while averaging signal intensities from probes that mapped to the same UG entry. For 10,876 UG IDs data was available for all 5 platforms. For the second subset, we mapped all probes to the Ensembl transcript database. There were 12,774 Ensembl transcripts that were interrogated by all 5 platforms.

**Figure 1:**
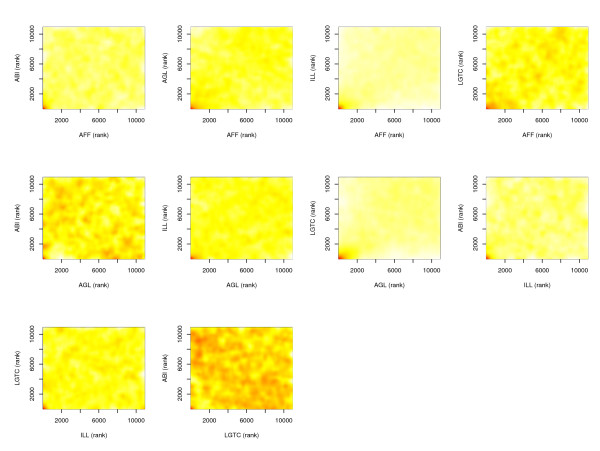
Scattersmooth plots of the correlation between the ranks (according to p values) of genes in the UG dataset of the 5 platforms. Red corresponds to denser areas, while yellow corresponds to non dense areas. The scattersmooth uses an algorithm for smoothing of two dimensional histograms with smoothed densities (26). This graph is more meaningful than a traditional scatter plot of the p values or of the -log p values, where the smallnumber of DEGs in our datasets originates graph blurred with thousands of overlapping dots and empty areas. Since the different signal to noise ratio is varying in the platforms and affects the statistics differently, plots of the ranks are more meaningful than plot of p values and statistics.

Results for the subset of genes with overlapping UG identifiers are reported in Table 1 and show the same trend already observed in the complete datasets. In Table 3 the overlaps in DEGs selected by each pair of platforms are reported. Two genes were selected by all 5 platforms (*Plac9*, *9230117N10Rik*). The 4 genes identified by ABI were selected on at least three other platforms. Overall, correspondence between platforms appears to be low. This is likely due to the use of a fixed statistical threshold. A higher correlation was found when evaluating the ranks of genes based on significance score. In Figure 1 the ranks for each gene are plotted for each pair of platforms. A scattersmooth function [34] is used for better visualization of the data cloud. As can be seen, in the area of the highly ranked genes (roughly from rank 1 – rank 200) there is a higher correlation between platforms than in the area of lower ranked genes. This is expected because only genes with significantly differential expression should be correlated while no correlation and complete scattering is expected for unchanged genes. We also considered the moderate t-statistics from the EBLRM which takes into account the direction of changes in the gene expression. The Pearson correlation coefficients (c_P_) of the t statistics within pair of platforms ranged between 0.10–0.47 (Table 3). Correlations between pairs of platforms belonging to the same type (one- or two-color) where higher than between those of different types, with c_P _= 0.47 between AFF – ILL and between AGL – LGTC. Given the fact that the correlations are calculated based on all genes of which the biggest majority does not change in expression, higher correlations are not to be expected.

**Table 3:** Overlap in DEGs in the UG subset (normal face) and Pearson correlation coefficients (bold face) between t statistics in each pair of platforms.

C_(*Pearson*)_\ # DEGs	AFF	ABI	ILL	AGL	LGTC
AFF	72	4	12	25	4
ABI	**0.26**	4	3	4	3
ILL	**0.47**	**0.22**	19	10	3
AGL	**0.18**	**0.16**	**0.17**	1594	19
LGTC	**0.11**	**0.10**	**0.11**	**0.47**	22

The results of the analysis of the Ensembl transcript-mapped overlapping probes were highly similar in terms of overlap (Table 1), and correlations of ranks and t-statistics (data not shown).

### Validation

Quantitative reverse transcription PCR (qRT-PCR) was used to validate the results of the different microarray platforms [see Additional file 2]. As expected the two genes found as DEGs by all five microarray platforms were confirmed to display differential expression. The fold-changes found by qRT-PCR were slightly higher than those found by any of the microarray platforms, confirming previous observations that ratios tend to be compressed in microarray experiments [21,23,35]. For 10 out of 11 tested genes that were significant (FDR<0.1) on at least two platforms, qRT-PCR experiments confirmed differential expression (Student's t-test: p < 0.05). *Lgals1*, that was found by AFF and ILL only, did not reach significance in the qRT-PCR experiment due to large variability in the wild-type group. We selected 15 genes (ranked from 8 to 719) that were found by AGL only covering the range from highly to lowly expressed genes, to ascertain whether the high number of genes selected by AGL was due to false positives. Eight out of these 15 genes were confirmed by qRT-PCR (p < 0.05), including *Spp1 *and *Camkk1*. These two genes were ranked among the top-350 genes on all platforms, except for *Camkk1 *on ABI. *Pip5k2a*, *Ttc3*, and *Acsl1 *were confirmed by qRT-PCR, but had an average ranking on the other platforms, and thus are truly found by AGL only. Of the 7 genes that were found by AGL only but could not be confirmed by qRT-PCR, *Gnb1l *and *Sgip1 *were border-line significant in the qRT-PCR experiment (p = 0.06). Interestingly, *Taf12*, although significant on AGL only, displayed very consistent fold-changes on the five microarray platforms (-1.08 to -1.12). Probably its fold-change was so low that it was hard to confirm by qRT-PCR.

### Gene set analysis

Analysis at the level of gene sets (as annotated in the Gene Ontology -GO- [36] and Kyoto Encyclopedia of Genes and Genomes -KEGG- [37] libraries) may reveal greater similarities between platforms than analysis at the level of individual genes, since different but functionally-related genes could give hints to aberrations in the same biological processes [38]. The Global Test was used to evaluate the differential regulation of gene sets [39]. This method is based on a model for predicting a response variable from the gene expression measurements of a set of genes. Unlike commonly overrepresentation test or Gene Set Enrichtment Analysis, it has optimal power in small sample size experiments and is able to identify gene sets where many genes display a small but consistent effect [40]. Furthermore, the test enables the control for array and dye effects, and produces easily interpretable p-values that can be compared across experiments.

**Figure 2:**
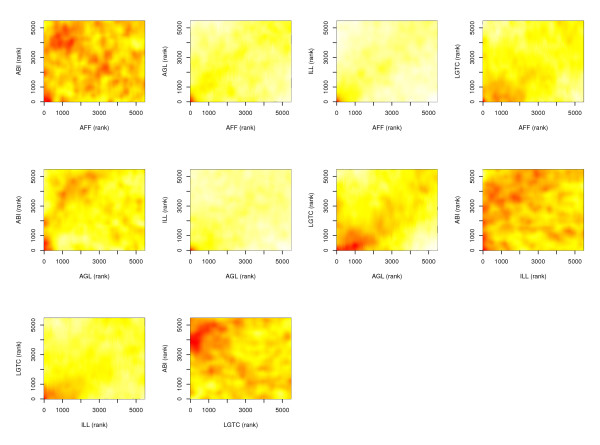
Scattersmooth plots between the ranks of the GO gene sets (according to Global Test p values).

We ranked the gene sets based on their Global Test significance and compared each pair of platforms (Figure 2). Like for the analysis of individual genes, the highly ranked gene sets showed good agreement across platforms. Again, the best correlations were observed between pairs of platforms of the same type: AFF-ILL (both one-color) and LGTC-AGL (both two-color) with Spearman correlation coefficients of 0.39 and 0.46 respectively. In agreement with the lower number of DEGs found by ABI, the results from ABI did not correlate well with those of the other platforms. Similar results were observed using the gene sets from KEGG (data not shown).

The list of gene sets that were consistently identified by at least three platforms is dominated by genes involved in GABAergic signaling (Table 4). *Gabra2*, found down-regulated on all platforms and confirmed by qRT-PCR [see Additional file 2], is the most influential gene in these gene sets. Different genes on different platforms contribute to the significance of these gene sets as a whole: e.g. *Chrna4 *(AFF, AGL, LGTC), *Chrna3 *(AGL), *Glra3 *(LGTC), *Glra4 *(ILL) for gene set GO:0004890. In general, this was due to near-background signals of these genes on most platforms.

**Table 4:** Gene sets highly ranked across platforms. For each platform, gene sets were ranked by their association with the phenotype under study, using the p-value from the global test. Displayed are those gene sets that rank highly in the majority of platforms; for GO sets, the sum of the highest three (out of five) ranks had to be below 100; for KEGG sets, the sum of the highest four (out of five) ranks had to be below 100. Columns 1 and 2 display GO/KEGG IDs and names of the gene sets (in parentheses: GO classification: BP = biological process; MF = molecular function). Columns 3 to 7 display the ranks of the gene sets for each of the platforms.

**GENE SET ID**	**Name**	**ABI**	**AFF**	**AGL**	**ILL**	**LGTC**
***GO***						
GO:0007214	gamma-aminobutyric acid signaling pathway (BP)	232	46	23	26	33
GO:0004890	GABA-A receptor activity (MF)	248	42	11	13	48
GO:0016917	GABA receptor activity (MF)	283	40	14	12	52
GO:0030594	neurotransmitter receptor activity (MF)	433	23	2	2	166
GO:0042165	neurotransmitter binding (MF)	474	22	1	1	174
GO:0006821	chloride transport (BP)	853	17	3	22	21
GO:0015698	inorganic anion transport (BP)	1022	7	21	33	76
GO:0005230	extracellular ligand-gated ion channel activity (MF)	1672	29	10	17	91
GO:0006820	anion transport (BP)	1801	8	33	34	98
GO:0015276	ligand-gated ion channel activity (MF)	1900	20	4	11	175
GO:0050900	leukocyte migration (BP)	13	898	12	69	1726
***KEGG***						
4080	Neuroactive ligand-receptor interaction	25	1	3	2	10
4512	ECM-receptor interaction	18	9	21	16	33
2010	ABC transporters – General	27	3	15	4	53
4660	T cell receptor signaling pathway	23	16	57	8	31
760	Nicotinate and nicotinamide metabolism	58	8	4	161	20
3030	DNA polymerase	9	11	12	24	138
4514	Cell adhesion molecules (CAMs)	39	13	23	17	115
900	Terpenoid biosynthesis	13	12	54	19	62
4640	Hematopoietic cell lineage	64	4	22	9	76

## Discussion

The aims of the present study were to compare the ability of different microarray platforms to detect differences in gene expression, when levels of regulation and numbers of regulated genes are low, and to investigate the influence of the platform in the biological interpretation of the results.

We show that even when gene expression differences between groups are small, several microarray platforms are able to consistently detect them. This is an important point, since in most previously published microarray platform comparisons, including the toxicogenomics MAQC study where biological replicates were analyzed, differences between samples analyzed where much larger than in our study [12,21,23-25]. The MAQC papers conclude that the cross-platform correlation is higher for fold-changes than for t-statistics. This is not true for our study. This apparent contradiction is because high fold-changes, which we simply do not have in our study, are more likely to be measured consistently, and contribute most to the Pearson correlation coefficient. Cross platform consistency in our study may compare favorably to another platform comparison study within a biological setting: Tan et al. reported a low agreement between 3 platforms (Affymetrix, Agilent, Amersham) in the analysis of the effect of serum withdrawal [14]. In their case, the amount of interrogated genes shared by all platforms was low. In our study, the number of common probes is bigger (N~ 12,000) and allows for more reliable comparisons since a bigger and possibly more representative set of probes is taken into consideration.

In contrast to other papers, we did not apply any filter to our data. In the reanalysis of the Tan dataset by Shi and collaborators [41] the authors claimed that the use of the unfiltered dataset gave a poor agreement between platforms, while restricting the analyses to a small filtered subset gives highly reproducible results. Even if several filters are commonly used, strict investigation on the possible bias introduced in the data because of the exclusion of genes has not been done. Since filters of the data may affect individual datasets differently, we have avoided using them in order to reflect the true unbiased gene expression signatures. The drawback is that the correlation measures are more affected by biological and technical noise.

The choice of the type of cut-off is still a matter of debate, and several authors suggested using a mixed cut-off of p-values and Fold Changes (FCs) [21,24]. However, even if a FC cut-off makes DEGs determination easier and from the technical point of view is more direct, it can eliminate the possibility of finding small differences in the data that are biologically interesting, as demonstrated in the current study (where only two genes showed a FC > 2). Furthermore, the FC statistics do not have the probabilistic characteristics guaranteed by theoretical conditions that allow to be sure about what the method does [42,43].

The degree of overlap between DEGs can be influenced by the overlap in interrogated and detectable transcripts as well as the method for matching of the probes. The overlap in interrogated transcripts was >75%, as expected for these whole genome microarray platforms. The overlap in probes with signal above background was also in the same range. However, by adding the two effects, one can explain as much as 50% of the difference between two platforms and this can be even more for home-spotted arrays were the numbers of detectable transcripts are often reduced due to local background problems. The overlap may be further reduced due to the interrogation of different splice variants that are mapped to the same UG identifier. The Ensembl transcript mapping accounts for alternatively spliced transcripts. However, the correlation between platforms in the Ensembl transcript-mapped dataset was, in our case, not higher than in the UG dataset. This could be due to complications in the mapping process: AFF probe sets sometimes cover more than one transcript, and for ABI oligonucleotide sequences were not provided but only 380 bp regions in which the probes were designed. Furthermore, there is considerable redundancy in the Ensembl transcript dataset due to multiple splice variants from the same gene being detected by all platforms, which may introduce biases in the downstream analyses. In this respect, the use of the recently released whole genome exon arrays for gene expression probably provides an attractive alternative, coping with such a problem.

AGL selected a ten-fold higher number of DEGs and significant gene sets than all other platforms. This is partly attributable to the high signal to noise level of this platform, as evident from the number of probes with signal higher than background. Still, this huge difference was unexpected and we investigated the behavior of the AGL data in more detail, and compared this with AFF and LGTC data using different approaches [see Additional file 1]. Briefly, the AGL log ratios show a bigger variability than AFF log intensities, measured by the *a posteriori *standard deviations. This difference remains after multiplying the variance of AFF intensities by the square root 2 in order to calculate the variance in the ratio between two samples. To check whether the doubled number of observations on the AGL were the cause for finding many more differentially expressed genes, we left AGL arrays out one by one and repeated the EBLRM analysis. The number of DEGs decreased steadily from 3,051 (10 arrays, 20 samples) to 649 (5 arrays, 10 samples). This is on the same order of magnitude as the number of DEGs of AFF (10 arrays, 10 samples, 130 DEGs), but still five times larger.

This suggests that the direct comparison of the wild-type and transgenic mouse samples on the same array drives the better performance, which is accordance with previous observations [13,33]. It argues against using either a common reference design or one-color protocols when comparing two groups of samples [21]. However, this does not explain the differences in performance between AGL and LGTC arrays. We found that AGL's technical replicates were much more reproducible than those of LGTC: Pearson correlation coefficients were 0.95–0.98 for AGL and 0.70–0.80 for LGTC, illustrating the differences in quality between commercial and home-spotted arrays. Overall, our study suggests that the differences in amount of DEGs found by the different platforms were mainly caused by differences in signal to noise ratios, and the numbers of observations between one and two-color platforms, when using the same number of arrays. Our qRT-PCR experiments validated differential gene expression in most cases, also for genes found by AGL only, indicating that these are not just false positives.

Our results illustrates once more that typical sample sizes used in microarray experiments, three samples per group, can be too small to enable reliable detection of subtle effects such as in this study. Even though using 5 samples per group still does not yield enough power for some platforms, it is possible to use our data as basis for estimation of sample size for the platforms considered. We are undergoing this work and the detailed analysis, beyond the scope of this paper, shall appear elsewhere. Our preliminary results confirm that AGL and AFF have comparable power, so the different outcomes observed by us are for the largest part due to the larger effective sample size involved in two-colour platforms design.

We investigated whether the power of the analysis could be enhanced by merging data from all five platforms in one statistical model. We applied an EBLRM on the UG subset and included samples, platforms and dye (only for the two-color arrays) as confounders. At an FDR of 0.1, 285 genes were selected (Table 1). Among these, most had been selected as DEGs by the individual platforms with the exception of 56 genes. However, we could not validate the differential expression of the top 5 of those genes by qRT-PCR, mainly due to large biological variation within groups. These genes seem to have been selected in the merged analysis due to the technical consistency on the microarray platforms allied to the larger pooled sample size.

This study also aims to elucidate the biological function of delta-DCLK-short expression in the hippocampus. Recent loss and gain of function studies strongly suggest involvement of the DCLK gene in neurogenesis, neuronal migration, vesicle transport, microtubule-directed retrograde transport, neurotransmission and apoptosis [28-30,44-46]. Thus, DEGs identified in this study may be involved in these processes. The present study focuses on comparison of different array platforms and therefore the results of the biological function will be discussed more extensively elsewhere (Schenk in preparation). However, it is interesting to note that the DEGs and the significant gene sets revealed by the different microarrays are biologically meaningful. For example, numerous gene sets related to GABA-ergic neurotransmission emerged as highly significant in 4 out of 5 platforms. Intriguingly, similarly as the DCLK gene, excitatory GABA signalling has been shown to control neurogenesis, neuronal migration and differentiation of neuroblasts [47,48]. DCLK-short expression starts postnatally around day 6, a timepoint that is characterized by a switch in excitatory GABAergic responses to inhibitory responses [49,50]. The added value of the use of different microarray platforms lies in the prioritization of the pathways for follow-up experiments. When analyzing data from a single platform, many spurious gene sets apparently not related to the biological process under study (e.g. chemotaxis) ranked highly, probably due to the relatively small expression differences observed. By comparing platforms, a biologically meaningful consensus could be distilled.

## Conclusion

The present study suggests that the choice of a platform can be mainly governed by practical and cost considerations. However, our data demonstrate that, given the much higher number of identified DEGs, commercial two-color platforms may be preferred when two groups with small differences in expression are to be compared. In these situations, a direct-comparison design helps to maximize signal-to-noise ratios in the ratios between the two groups through minimization of the array effect and the possibility for more replicates with the same number of arrays. Since we performed this study with a clear underlying biological question, we could demonstrate that there was agreement across platforms in the perturbed biological processes identified. Consistency between platforms helped to prioritize biological processes relevant for the biological question under study. The relevant gene sets were detected with an only partly overlapping set of genes. Our data indicate that when analyzing samples with only subtle differences in gene expression the use of two different platforms might be more rewarding than increasing the number of replicates on the same platform.

## Methods

### MICE

5 Wild-type male C57/BL6j and 5 transgenic male mice over-expressing DCLK-short with a C57/BL6j background were individually housed 7 days prior to the start of the experiment. Animals were housed under standard conditions, 12h/12h light/dark cycle and had access to food and water ad libitum. Wild-type (N = 5) and transgenic (N = 5) tissue samples were collected by taking the brain from the skull and quickly dissecting out both hippocampi. Dissection was performed at 0°C to prevent degradation of RNA. Hippocampi were put directly in pre-chilled tubes containing Trizol reagent (Invitrogen Life Technologies, Carlsbad, CA, USA). All animal treatments were approved by the Leiden University Animal Care and Use Committee (UDEC# 01022).

### RNA extraction

After transfer to ice-cold Trizol, hippocampi were homogenized using a tissue homogenizer (Salm&Kipp, Breukelen, The Netherlands) and total RNA was isolated according to the manufacturer's protocol. After precipitation, RNA was purified with Qiagen's RNeasy kit with on-column DNase digestion. The quality of the RNA was assessed with the RNA 6000 Labchip kit in combination with the Agilent 2100 Bioanalyzer (Agilent Technologies, Palo Alto, CA, USA), using the Eukaryote Total RNA Nano assay according to the manufacturer's instructions. Total RNA was amplified using Ambion's MessageAmp kit, with incorporation of modified nucleotides (biotin-16-UTP (AFF, ILL), aminoallyl-UTP (AGL, LGTC), DIG-UTP (ABI)). For AGL and LGTC, aminoallyl-cRNA was coupled to Cy3 or Cy5 monoreactive dyes (GE Healthcare).

### Experimental design

Labelled cRNAs of 5 individual wild-type and 5 transgenic mice were hybridized on 5 different microarray platforms (Table 1): Applied Biosystem (ABI), Affymetrix (AFF), Agilent (AGL), Illumina (ILL), and home-spotted glass microarrays containing the 22K mouse Sigma-Compugen collection generated at the Leiden Genome Technology Center (LGTC). Ten microarrays were used for each platform. For the one-color platforms (ABI, AFF, ILL), each individual RNA was hybridized to one microarray. A direct design was used for hybridization of the two-color arrays (AGL, LGTC), *i.e*. each microarray was hybridized with two RNA samples from different groups. All samples were hybridized once in Cy3 and once in Cy5. Dye-swapped hybridizations were done with non-identical sample pairs [see Additional file 3].

### Quantitative RT-PCR

Quantitative RT-PCRs were done on the Lightcycler480 (Roche), using the universal probe library (UPL, Roche) or SYBR-Green (when amplification efficiencies with UPL were below 90%). The RNA samples used for validation were the same as in the microarray experiments. Each cDNA was analyzed in quadruplicate, after which the average threshold cycle (Ct) was calculated per sample. Differential expression was evaluated with a Student's *t*-test, considering the 5 biological replicates in each group.

### Mapping

Two approaches were used to obtain an overlapping gene set that was measured on each platform. The first is based on the annotation provided by the manufacturer, while the second is an in-house performed probe sequence-based annotation.

1. GenBank accession numbers that were used for the design of the microarray probes were used for querying the Mus musculus Unigene (UG) database build #151. All UG IDs that occurred at least once on each platform were included in the UG set (N = 10,876). With this UG set, a UG dataset was created for each platform by extracting the expression values for the relevant probes. When multiple probes were present for the same UG ID, the average of the signal of the probes was used as expression value.

2. For AGL, LGTC, and ILL, probe sequences provided by the manufacturer were directly used for annotation. For AFF, the 11 probe sequences in a probe set were concatenated, after removal of potential overlap. ABI did not reveal the exact probe sequences but a 380 bp region, in which the probes were located. Gmap [51] was used for alignment of the sequences to the Ensembl mouse genome sequence (build NCBIM34). Hits with a match score higher than 0.9 (matches – gaps/query size [52]) were considered genuine matches. Chromosomal start and end positions of the hits were compared to the exon positions in the Ensembl database (version 37.34e). Subsequently, the Ensembl Transcript database was queried with only the exons that matched (part of) the probe sequence. Only transcripts with a match score >0.9 on all 5 platforms were included the Ensembl transcript set (N = 12,744). When multiple probes were present for the same transcript, the average of the signal of the probes was used as expression value.

Based on each of the above overlapping gene sets, a dataset was created for each platform, which was analyzed separately [for UG: see Additional file 4]. For completeness, the complete datasets (including also the non overlapping probes) were also analyzed in parallel.

### Preprocessing procedures

The quality of the arrays was assessed by visual inspection of the raw images and pairwise MA-plots. No arrays were excluded from the analysis since the variance on the log-ratios was comparable between arrays. For the ABI platform, we observed differences in the signal distribution between two batches of arrays hybridized on two different days, for the other platform no quality problems were observed. Each dataset was loaded into the R environment directly as a raw data matrix (for ABI and ILL) or using the limma package (AFF, AGL, and LGTC). No background correction was applied to the two-color microarray platforms since the background correction increased noise levels in the low intensity range considerably. For AFF analysis, only perfect match probes were taken into account and probesets were summarized with the "median polish" method. The data from the one-color platforms were normalized with variance stabilization and normalization function implemented in the vsn package [53]. From all the normalization methods tested, vsn was most robust, whereas the performance of alternative normalization algorithms was more platform-dependent. Two-color arrays were normalized with loess [54] since vsn normalization did not correct all the intensity-dependent non-linear behaviour in the data. Raw and normalized data are available in GEO under series GSE8349.

### Present calls

For Affymetrix chips, probes were said to be present when the MAS5.0 present call algorithm called the probe "P" (present) on all 10 arrays. For the other platforms, probes were said to be present when their signal intensity was above the signal from the lowest 95% of platform-specific negative control probes on all 10 arrays. For the two colour platforms, this requirement was imposed on the intensities of both the green and the red channel. Lists of present probes for each platform were then mapped to the ENSEMBL transcript database to generate a list of unique ENSEMBL transcript IDs with detectable expression.

### Statistical analyses

#### Determination of Differentially Expressed Genes (DEGs)

Each dataset was analyzed for determination of DEGs using an Empirical Bayes Linear Regression Model (EBLRM). The following models were used for this purpose:

1) one-color datasets

y_i _= α_i _+ β_i _group + ε_i_

2) two-color datasets- log ratios

w_i _= α_i _+ ε_i_

3) two-color datasets-intensities

y_i _= α_i _+ β_i _group + γ_i _dye + δ_i _array + ε_i_

where i is the i^th ^item of the datasets, y_i _is the intensity signal, w_i _is the log ratios of the signal in Cy3 dye vs the Cy5 dye; α_i_, β_i_, γ_i_, δ_i_, ε_i _were the coefficients of the intercept, group (transgenic vs. wild type), dye (Cy5 vs. Cy3 – only for two-color arrays), array (only for two-color arrays), and error terms, respectively. All the effects were considered to be random. DEGs were defined as the probes for which the β_i _were significantly different from 0, since β_i _is the estimate for the group (wild-type or transgenic) effect. Analysis were performed with the limma package [32], using the lmFit function. P values were adjusted for multiple testing using the False Discovery Rates (FDR) method suggested by Benjamini and Hochberg [55]. FDR not greater than 10% was considered as statistically significant. Numbers and percentages of overlapping items in the list of DEGs among the 5 platforms were calculated.

Genes of UG and Ensembl were ordered by their p values obtained from the EBLRM and their Spearman correlation coefficients (c_S_) were calculated for pairs of platforms. Pearson correlation coefficients (c_P_) were calculated to quantify the correlation between the statistics produced by the EBLRM in the 5 overlapping datasets.

#### Gene set analysis

The association between the multiple functionally-related genes belonging to the same gene sets (according to the GO [36] and KEGG [37] libraries) and the group was assessed using the Global Test [39]. A logistic model with a gamma p-value estimating method was used for all platforms. For the two-color arrays, intensities were extracted and a model including array and dye effects as confounders was used. Gene sets were ordered by their p values obtained from the global test and Spearman correlation coefficients were calculated for pairs of platforms. Multiple testing was corrected via the FDR method [55]. FDR not greater than 10% was considered as statistically significant.

#### Two-color platforms data analysis

Analyses of the two-color platforms data were done using log ratios per array, whenever possible. However, for the gene set analysis and for the analysis of the merged datasets separate channel intensities were needed. These were then extracted from the raw data, normalized using vsn and, to account for technical variability, the analysis model also included array and dye as confounders.

#### Software

All the analyses were performed using R software environment [56] version 2.3.2 and BioConductor [57] packages vsn [53], loess [54], multtest [58], Affy [59], globaltest [39], limma [32], AnnBuilder [60] and the function scattersmooth [34].

When metadata packages were available at the BioConductor website (in our case, for AGL and AFF platforms), we used them for the annotation. Otherwise (for ABI, ILL, and LGTC) the annotation packages were produced using the AnnBuilder package in R [60].

## Abbreviations

ABI: Applied Biosystems; AFF: Affymetrix; AGL: Agilent; DCLK: δC-doublecortin-like kinase; DEG: Differentially expressed genes; EBLRM: Empirical Bayes Linear Regression Model; FC: Fold Change; FDA: Food and Drug Administration; FDR: False discovery rate; GABA: Gamma amino butyric acid; GO: Gene Ontology; KEGG: Kyoto Encyclopedia of Genes and Genomes; ILL: Illumina; LGTC: Home-spotted LGTC arrays; MAQC: MicroArray Quality Control Consortium; qRT-PCR: Quantitative reverse transcription PCR; UG: Unigene.

## Authors' contributions

PP conducted all the statistical analyses and drafted the manuscript, PAtH contributed to the study design, the data analysis and the drafting of the manuscript, EV worked on the biological interpretation of the results, GJS isolated the RNA and contributed to the biological interpretation, RHAMV performed the validation via qRT-PCR, YA was responsible for the microarray experiments, MdH and RK reannotated the microarray probe sequences, GJBvO corrected the manuscript, JdD and JB helped in the setting up of the experiments and contributed to the interpretation of the results, RM was mainly involved in the supervision of the statistical analyses and statistical interpretation of results.

## Supplementary Material

Additional file 1 suppl.material.pedotti. contains a more detailed comparison of the performance of AGL and AFF microarrays. Click here for file

Additional file 2 table.S1.pedotti. contains a list of the genes selected for the validation with qRT-PCR and its results. Click here for file

Additional file 3 table.S2.pedotti. contains the hybridization design for the two color arrays (AGL and LGTC). Click here for file

Additional file 4 all.data.UG. contains the expression data of all the 5 platforms for the subset of genes with overlapping UG identifiers. Click here for file
