# Supplementary material for: Can subtle changes in gene expression be consistently detected with different microarray platforms?
Source: BMC Genomics. 2008 Mar 10;9:124. doi: 10.1186/1471-2164-9-124 (PMC2335120; doi:10.1186/1471-2164-9-124)

Supplemental Material

Detailed analyses were performed to investigate the high sensitivity of AGL compared with the others platforms, especially AFF (that is one of the most widely used platforms) and LGTC (that has a two color design)

First of all, we compared the data of AGL and AFF, from different points of view. Since AGL is selecting so many DEGs, we expected that the distributions of the *a posterior* standard deviations used in the EBLRM to be smaller than the one in the AFF data. Surprisingly, the log-ratios from AGL displayed more variability than the log intensities of AFF (Fig. S1). This suggests that the ability of AGL to detect so many DEGs is not related to a smaller variance.

To investigate if the assumptions of the EBLRM were violated, we performed an analysis applying EBLRM on 1,000 datasets obtained by random permutations of the sample labels. The distributions of the empirical p values obtained from the permutations is similar to the ones obtained from the real data (fig s2): in the AGL there is a higher increased amount of low p values than in AFF, showing that the effect in that dataset is bigger. Results from the permutations analysis suggest that the assumptions of the EBLRM were not violated.

The reason for the higher sensitivity of AGL could be driven by bigger sample size due to the two color design . To investigate this, we applied the EBLRM to the AGL data, after sequentially leaving out up to 5 arrays, trying to maintain the balance of the dye and the presence of all the individual samples. The number of DEGs decreases steadily from 3,051 (10 arrays, 20 samples) to 649 (5 arrays, 10 samples). This is on the same order of magnitude as the number of DEGs more similar to the AFF one (10 arrays, 10 samples, 130 DEGs), but still five times larger.

Thus, the two color design yelding to a double sample size seems to play an important role in the higher sensitivity of AGL. However, the other two color platform (LGTC) was selecting much fewer DEGs. We investigated the difference in terms of noise in these two platforms. For this purpose, we plotted the data of the same sample in the two channels (fig s3). The correlation between the same samples were higher in AGL (0.95-0.98) than in LGTC (0.70-0.80).

These results suggest that the increased power of AGL in selecting more items is not merely due to technical effects and the bigger sample size but also to a larger precision of the platforms itself.


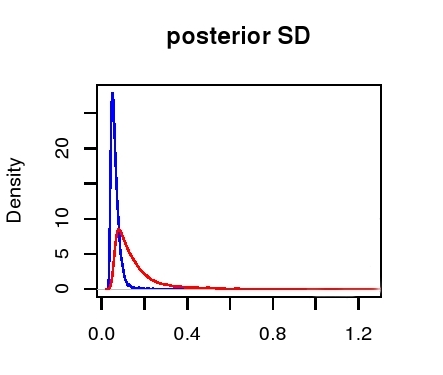
Figure s1. Distributions of the densities of the *a posterior* standard deviations used in the EBLRM, in AGL data (red line) and in AFF (blue line).

Figure s2. Density of the empirical p values, in AGL (red line) and AFF (black line) calculated applying the EBLRM to 1000 permutations of the labels in the original dataset.


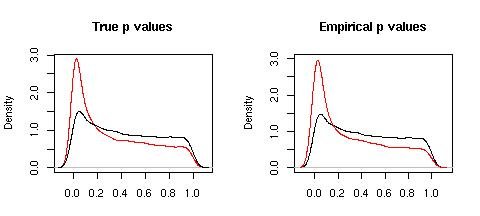


Figure s3. Example of correlation between signal of the same sample in the two single channel (red vs green) in the AGL intensities data (left panel) and in LGTC (right panel) for the WT3 mouse.


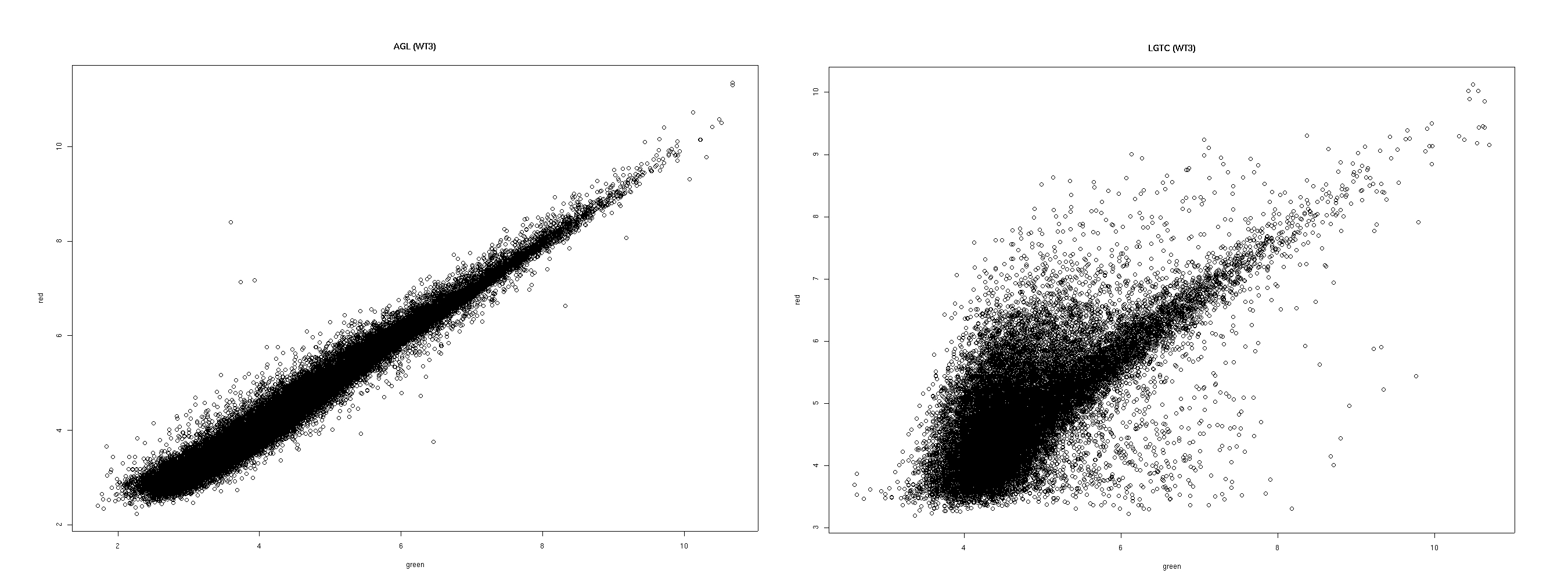

Supplement: Additional file 1 — suppl.material.pedotti. contains a more detailed comparison of the performance of AGL and AFF microarrays. [file 1471-2164-9-124-S1.doc]
