# Supplementary material for: Can subtle changes in gene expression be consistently detected with different microarray platforms?
Source: BMC Genomics. 2008 Mar 10;9:124. doi: 10.1186/1471-2164-9-124 (PMC2335120; doi:10.1186/1471-2164-9-124)
Supplement: Additional file 2 — table.S1.pedotti. contains a list of the genes selected for the validation with qRT-PCR and its results. [file 1471-2164-9-124-S2.doc]

Table S1. Validation of differential expression by qRT-PCR. Two genes were selected as DEGs in all the 5 platforms; 2 in 4 platforms; 4 in 3 platforms; 5 in 2 platforms; 15 in AGL only. The first column contains the gene symbol; the second column reports the platforms which detect the gene as DEG; the columns 3- 8 reports the mean ^2^log ratio for the different microarray platforms and the qRT-PCR assay; column 9 reports the level of significance in the qRT-PCR data from the Student’s t-test; in bold characters significant p values (p < 0.05).

| Symbol | Significance at FDR 0.1 | ABI | AFF | AGL | ILL | LGTC | qRT-PCR | Pval qRT-PCR |
| --- | --- | --- | --- | --- | --- | --- | --- | --- |
| Plac9 | ABI, AFF, AGL, ILL, LGTC | 1.17 | 1.09 | 0.99 | 0.79 | 0.3 | 1.94 | **0.0000** |
| 9230117N10Rik | ABI, AFF, AGL, ILL, LGTC | 0.54 | 0.37 | 0.58 | 0.35 | 0.31 | 0.82 | **0.0001** |
| Gabra2 | ABI, AFF, AGL, ILL | -0.95 | -0.95 | -0.57 | -0.48 | -0.47 | -1.38 | **0.0000** |
| Fabp7 | ABI, AFF, AGL, LGTC | -0.48 | -0.55 | -0.8 | 0 | -0.49 | -0.69 | **0.0076** |
| Mthfd2 | AFF, AGL, ILL | 0.47 | 0.18 | 0.09 | 0.24 | -0.01 | 0.55 | **0.0000** |
| Chac1 | AFF, AGL, ILL | 0.43 | 0.35 | 0.13 | 0.36 | 0.01 | 0.55 | **0.0000** |
| Bok | AFF, AGL, ILL | 0.29 | 0.36 | 0.42 | 0.37 | 0.11 | 0.69 | **0.0042** |
| Atf4 | AFF, AGL, LGTC | 0.19 | 0.17 | 0.59 | 0.19 | 0.19 | 0.42 | **0.0002** |
| Trem2 | AFF, ILL | 0.24 | 0.23 | 0.06 | 0.26 | -0.02 | 0.37 | **0.0023** |
| Lynx1 | AFF, AGL | 0.03 | -0.21 | -0.34 | -0.11 | -0.3 | -0.2 | **0.0149** |
| Rasl11b | AFF, AGL | -0.08 | -0.2 | -0.32 | -0.12 | -0.17 | -0.54 | **0.0386** |
| Lgals1 | AFF, ILL | 0.19 | 0.35 | -0.03 | 0.25 | 0.15 | 0.24 | 0.2977 |
| Arhgdig | AGL, ILL | -0.09 | -0.16 | -0.5 | -0.18 | -0.26 | -0.51 | **0.0013** |
| Gnb1l | AGL | -0.01 | -0.04 | 0.16 | -0.03 | -0.01 | -0.24 | 0.0571 |
| Cenpa | AGL | -0.12 | -0.03 | -0.15 | -0.04 | 0.02 | -0.35 | **0.0289** |
| Cask | AGL | 0.06 | -0.01 | 0.2 | -0.03 | 0.1 | 0.07 | 0.3989 |
| Irgm | AGL | 0.26 | 0.07 | 0.18 | 0.07 | 0.11 | 0.41 | **0.0144** |
| Sgip1 | AGL | 0.14 | 0.02 | 0.32 | 0.01 | 0.02 | 0.24 | 0.0608 |
| Acsl1 | AGL | 0.15 | 0.04 | 0.32 | 0.07 | 0.03 | 0.43 | **0.0330** |
| Ttc3 | AGL | -0.22 | -0.07 | 0.4 | -0.01 | 0.2 | 0.35 | **0.0033** |
| Pip5k2a | AGL | 0.24 | 0.05 | 0.28 | 0.11 | 0.06 | 0.29 | **0.0418** |
| Gng3 | AGL | 0.16 | -0.13 | -0.57 | 0.04 | -0.21 | -0.19 | **0.0095** |
| Taf12 | AGL | -0.12 | -0.12 | -0.18 | -0.11 | -0.13 | -0.09 | 0.1308 |
| Camkk1 | AGL | 0.07 | 0.12 | 0.24 | 0.11 | 0.22 | 0.2 | **0.0115** |
| Spp1 | AGL | 0.73 | 0.26 | 0.43 | 0.27 | 0.19 | 0.83 | **0.0128** |
| Efcbp2 | AGL | -0.1 | -0.1 | -0.22 | -0.06 | -0.18 | -0.11 | 0.1856 |
| Kcnab3 | AGL | -0.12 | 0.02 | -0.2 | -0.01 | 0 | -0.01 | 0.7773 |
| Arc | AGL | -0.17 | -0.06 | -0.3 | -0.07 | -0.12 | -0.31 | 0.1319 |
