# Supplementary material for: Can subtle changes in gene expression be consistently detected with different microarray platforms?
Source: BMC Genomics. 2008 Mar 10;9:124. doi: 10.1186/1471-2164-9-124 (PMC2335120; doi:10.1186/1471-2164-9-124)
Supplement: Additional file 3 — table.S2.pedotti. contains the hybridization design for the two color arrays (AGL and LGTC). [file 1471-2164-9-124-S3.doc]

Table S2. Hybridization design for two color platforms (AGL and LGTC).

| Array | Individual Cy3 | Individual Cy5 |
| --- | --- | --- |
| 1 | WT1 | dC4 |
| 2 | WT3 | dC5 |
| 3 | WT4 | dC2 |
| 4 | WT5 | dC3 |
| 5 | WT6 | dC1 |
| 6 | dC1 | WT5 |
| 7 | dC2 | WT1 |
| 8 | dC3 | WT4 |
| 9 | dC4 | WT3 |
| 10 | dC5 | WT6 |
